# Supplementary material for: Electrophysiology-Guided Genetic Characterisation Maximises Molecular Diagnosis in an Irish Paediatric Inherited Retinal Degeneration Population
Source: Genes (Basel). 2022 Mar 29;13(4):615. doi: 10.3390/genes13040615 (PMC9033125; doi:10.3390/genes13040615)
Supplement: Supplementary file 1 [file genes-13-00615-s001.zip › genes-1600529-supplementary.pdf]

genes

Article

Electrophysiology-Guided Genetic Characterisation Maximises Molecular Diagnosis in an Irish Paediatric Inherited Retinal Degeneration Population

Julia Zhu [1,*], Kirk A. J. Stephenson [1,2], Adrian Dockery [3], Jacqueline Turner [1], James J. O'Byrne [1], Susan Fitzsimon [2], G. Jane Farrar [4], D. Ian Flitcroft [2] and David J. Keegan [1,2]

Table S1. Full genetic result.

Patient No	Pedigree No	Age	Gender	Phenotype	Genotype	Gene	Inheritance	Variant 1	ACMG Classification	Variant 2	ACMG Classification	Variant 3	ACMG Classification
1	1	9	M	ESORD	AS	*ALMS1*	AR	c.1199_1205del, p.(Thr400Lysfs*11)	5	c.8456_8817del, p.(Thr2819Argfs*29)	5		
2	2	5	M	BVMD	BVMD	*BEST1*	AD	c.874G>A, p.(Glu292Lys)	5				
3	3	15	M	BVMD	BVMD	*BEST1*	AD	c.472C>T, p.Arg158Cys	5				
4	3	18	F	BVMD	BVMD	*BEST1*	AD	c.472C>T, p.Arg158Cys	5				
5	4	2	M	CD	ACHM	*CNGA3*	AR	c.667C>T, p.(Arg223Trp)	5	c.667C>T, p.(Arg223Trp)	5		
6	5	2	F	CD	ACHM	*CNGB3*	AR	c.1148del, p.(Thr383Ilefs*13)	5	c.1148del, p.(Thr383Ilefs*13)	5		
7	6	6	M	CD	ACHM	*CNGB3*	AR	c.1148del, p.(Thr383Ilefs*13)	5	c.1148del, p.(Thr383Ilefs*13)	5		
8	7	11	M	CD	-	negative	-						
9	8	13	F	CD	ACHM	*CNGB3*	AR	c.1148del, p.(Thr383Ilefs*13)	5	c.1148del, p.(Thr383Ilefs*13)	5		
10	9	13	F	CD	ACHM	*CNGB3*	AR	c.1578+1G>A	5	c.1006G>T, p.(Glu336*)	5		
11	10	14	F	CD	ACHM	*CNGA3*	AR	c.1228C>T, p.(Arg410Trp)	5	c.1228C>T, p.(Arg410Trp)	5		
12	10	18	M	CD	ACHM	*CNGA3*	AR	c.1228C>T, p.(Arg410Trp)	5	c.1228C>T, p.(Arg410Trp)	5		
13	11	16	M	CD	-	negative	-						
14	12	16	M	CD	-	negative	-						
15	13	17	M	CD	ACHM	*PDE6H*	AR	c.199G>T, p.(Glu67*) †	3	c.199G>T, p.(Glu67*) †	3		
16	14	17	M	CD	CD	*KCNV2*	AR	c.(1356+1_1357-1)_(*1_?)del	5	c.1637A>C, p.(*546Serext*60)	4		
17	15	17	F	CD	-	negative	-						
18	16	18	F	CD	ACHM	*CNGB3*	AR	c.1148del, p.(Thr383Ilefs*13)	5	c.1148del, p.(Thr383Ilefs*13)	5		
19	17	19	F	CD	ACHM	*CNGB3*	AR	c.1148del, p.(Thr383Ilefs*13)	5	c.1148del, p.(Thr383Ilefs*13)	5		
20	18	19	M	CD	-	negative	-						
21	19	19	F	CD	CD	*KCNV2*	AR	c.778A>T, p.Lys260Ter	5	c.958C>T, p.Arg320Cys	5		
22	20	7	F	CSNB	-	negative	-						

23	21	12	M	CSNB	CSNB	*NYX*	XL	c.856del, p.(Asp286Thrfs*62)	5		
24	21	14	M	CSNB	CSNB	*NYX*	XL	c.856del, p.(Asp286Thrfs*62)	5		
25	22	17	M	CSNB	CSNB	*NYX*	XL	c.602C>A, p.(Ser201*) †	5		
26	23	17	F	CSNB	CSNB	*TRPM1*	AR	c.215A>G, p.(Tyr72Cys)	5	c.3004A>T, p.(Ile1002Phe)	4
27	24	17	M	CSNB	CSNB	*CACNA1F*	XL	c.2632_2634delinsATCCTCAGCAGTGTGTCCCCAGCA, p.(Leu878delinsIleLeuSerSerValSerProAla) †	3		
28	25	23	M	CSNB	CSNB	*NYX*	XL	c.460C>T, p.(Leu154Phe) †	3		
29	26	27	F	CSNB	CSNB	*TRPM1*	AR	c.296T>C, p.(Leu99Pro)	5	c.215A>G, p.(Tyr72Cys)	5
30	26	28	M	CSNB	CSNB	*TRPM1*	AR	c.296T>C, p.(Leu99Pro)	5	c.215A>G, p.(Tyr72Cys)	5
31	27	1	M	LCA	LCA	*AIPL1*	AR	c.277-2A>G	5	c.553G>A, p.(Gly185Arg)	3
32	28	1	M	LCA	LCA	*RPE65*	AR	c.886dup, p.Arg296Lysfs*7	5	c.130C>T, p.Arg44*	5
33	28	4	M	LCA	LCA	*RPE65*	AR	c.886dup, p.Arg296Lysfs*7	5	c.130C>T, p.Arg44*	5
34	29	2	M	LCA	LCA	*CRX*	AD	c.851del, p.(Asp284Alafs*87)	4		
35	30	2	M	LCA	LCA	*AIPL1*	AR	c.834G>A, p.(Trp278*)	5	c.784G>A, p.(Gly262Ser)	4
36	31	2	M	LCA	LCA	*CEP290*	AR	c.1781T>A, p.(Leu594*)	5	c.2888A>G, p.(Glu963Gly) †	3
37	31	13	F	LCA	LCA	*CEP290*	AR	c.1781T>A, p.(Leu594*)	5	c.2888A>G, p.(Glu963Gly) †	3
38	32	8	M	LCA	LCA	*CRB1*	AR	c.2230C>T, p.(Arg744*)	5	c.1745T>C, p.(Leu582Ser) †	4
39	33	8	F	LCA	LCA	*RDH12*	AR	c.883C>T, p.(Arg295*)	5	c.910T>C, p.(Trp304Arg)	4
40	34	9	F	LCA	-	negative	-				
41	35	6	M	RCD	-	negative	-				
42	36	11	M	RCD	RCD	*RP2*	XL	c.700G>T, p.(Glu234*)	4		
43	37	11	F	RCD	RCD	*PRPF8*	AD	c.6950_6951del, p.(Phe2317Cysfs*67) †	4		

44	38	13	F	RCD	-	negative	-						
45	39	15	M	RCD	RCD	*RPGR*	XL	c.646G>T, p.Glu216Ter	4				
46	39	18	M	RCD	RCD	*RPGR*	XL	c.646G>T, p.Glu216Ter	4				
47	40	15	M	RCD	RCD	*RPGR*	XL	c.2894del, p.Glu965Glyfs*124	5				
48	40	18	M	RCD	RCD	*RPGR*	XL	c.2894del, p.Glu965Glyfs*124	5				
49	41	16	M	RCD	RCD	*CNGB1*	AR	c.413-1G>A	5	c.346C>T, p.(Gln116*) ✝	5		
50	42	16	F	RCD	RCD	*PRPF31*	AD	c.240_24insT, p.Met81TyrfsTer9	4				
51	42	22	M	RCD	RCD	*PRPF31*	AD	c.240_24insT, p.Met81TyrfsTer9	4				
52	43	18	M	RCD	RCD	*RHO*	AD	c.533A>G, p.(Tyr178Cys)	5				
53	44	19	F	RCD	RCD	*RPGR*	XL	c.2777_2778del, p.Glu926Glyfs*152 ✝	5				
54	45	20	F	RCD	RCD	*IMPDH1*	AD	c.527T>C, p.(Ile176Thr) ✝	3				
55	46	20	F	RCD	-	negative	-						
56	47	21	M	RCD	RCD	*RLBP1*	AR	c.466C>T, p.(Arg156*)	5	c.13-1G>C ✝	4		
57	48	11	F	Usher	Usher	*MYO7A*	AR	c.4502_4503del, p.(Val1501Glyfs*2)	4	c.4502_4503del, p.(Val1501Glyfs*2)	4		
58	49	20	F	BBS	BBS	*BBS1*	AR	c.1169T>G, p.(Met390Arg)	5	c.1169T>G, p.(Met390Arg)	5		
59	50	14	F	STGD	STGD	*ABCA4*	AR	c.5882G>A, p.(Gly1961Glu)	5	c.4363T>C, p.(Cys1455Arg)	5		
60	51	15	M	STGD	STGD	*ABCA4*	AR	c.4139C>T, p.Pro1380Leu	5	c.4469G>A,p.Cys1490Tyr	5		
61	52	21	M	STGD	STGD	*ABCA4*	AR	c.6658C>T, p.Gln2220Ter	5	c.550_551delinsCG, p.Ser184Arg	4		
62	53	22	M	STGD	STGD	*ABCA4*	AR	c.4139C>T, p.Pro1380Leu	5	c.6079C>T,p.Leu2027Phe	5		
63	54	26	F	STGD	STGD	*ABCA4*	AR	c.161G>A, p.Cys54Tyr	5	c.1317G>A, p.Trp439Ter	4	c.5908C>T, p.Leu1970Phe	4
64	55	7	M	XLRS	XLRS	*RS1*	XL	c.413C>A, p.(Thr138Asn)	5				
65	55	10	M	XLRS	XLRS	*RS1*	XL	c.413C>A, p.(Thr138Asn)	5				
66	55	13	M	XLRS	XLRS	*RS1*	XL	c.413C>A, p.(Thr138Asn)	5				

67	55	14	M	XLRS	XLRS	*RS1*	XL	c.413C>A, p.(Thr138Asn)	5
68	56	13	M	XLRS	XLRS	*RS1*	XL	c.329G>A, p.Cys110Tyr	5
69	56	21	M	XLRS	XLRS	*RS1*	XL	c.329G>A, p.Cys110Tyr	5
70	57	14	M	XLRS	XLRS	*RS1*	XL	c.304C>T, p.Arg102Trp	5

ACHM - Achromatopsia. AS – Alström Syndrome. BBS - Bardet Biedl Syndrome. BVMD – Best Vitelliform Macular Dystrophy. CD – Cone Dystrophy. CSNB – Congenital Stationary Night Blindness. EOSRD - Early-onset Severe Retinal Dystrophy. LCA – Leber Congenital Amaurosis. RCD – Rod Cone Dystrophy. STGD – Stargardt Disease. XLRS – X-linked Retinoschisis. † novel variant.

Table S2. Additional findings detected.

Patient No.	Pedigree No.	Additional Finding Gene	Variant	ACMG Classification
5	4	*OAT*	c.952G>A, p.(Glu318Lys)	5
6	5	*ABCA4*	c.5908C>T, p.(Leu1970Phe)	3
9	8	*SLC25A46*	c.746G>A, p.(Gly249Asp)	3
10	9	*TRPM1*	c.3743G>A, p.(Arg1248Gln)	3
11	10	*POMGNT1*	c.244C>G, p.(Leu82Val)	3
		HGSNAT	c.1129-3C>G	3
13	11	*RDH12*	c.648_658+20del	4
15	13	*USH2A*	c.13339A>G, p.(Met4447Val)	5
17	15	*RTN4IP1*	c.637G>A, p.(Ala213Thr)	3
19	17	*TRPM1*	Whole gene deletion	5
20	18	*ABCA4*	c.4253+43G>A	5
		CRB1	c.498_506del, p.(Ile167_Gly169del)	5
22	20	*TRPM1*	c.1461G>C, p.(Met487Ile)	3
		EYS	c.2739G>T, p.(Arg913Ser)	3
		BBS4	c.333-2A>C	4
24	21	*HGSNAT*	c.1150C>T, p.(Arg384*)	5
25	22	*TMEM231*	c.248C>A, p.(Ser83*)	4
		KCNV2	c.1292C>T, p.(Ser431Phe)	3
27	24	*PNPLA6*	c.297-4_297-2del	3
28	25	*CNGA1*	c.1166C>T, p.(Ser389Phe)	5
31	27	*ABCC6*	c.3421C>T, p.(Arg1141*)	5
		C8orf37	c.155+5A>T	3
		VPS13B	c.5507C>G, p.(Pro1836Arg)	3
		VPS13B	c.7996C>T, p.(Arg2666Cys)	3
33	28	*RBP3*	c.2270T>G, p.(Val757Gly)	3
		PDE6A	c.2144T>C, p.(Met715Thr)	3
35	30	*PHYH*	c.581C>T, p.(Thr194Met)	3
37	31	*RLBP1*	c.647G>A, p.(Arg216Gln)	3
38	32	*TTC21B*	c.3459+1G>A	4
		CSPP1	c.167_168del, p.(Lys56Serfs*6)	5
		CA4	c.162C>G, p.(Ile54Met)	3
39	33	*USH2A*	c.12457G>A, p.(Ala4153Thr)	4
		ROM1	c.339dup, p.(Leu114Alafs*18)	3
40	34	*TRPM1*	c.382G>T, p.(Ala128Ser)	3
		CEP250	c.493-1G>A	3
41	35	*LRP2*	c.2800G>A, p.(Gly934Ser)	3
42	36	*TCTN3*	c.338_341del, p.(His113Argfs*13)	4
44	38	*ABCA4*	c.6320G>A, p.(Arg2107His)	5
		ABCA4	c.618C>G, p.(Ser206Arg)	3
		TTLL5	c.1450C>T, p.(Arg484Cys)	3
48	40	*FAM161A*	c.1309A>T, p.(Arg437*)	5
		GPR179	c.2706_2707dup, p.(Pro903Hisfs*67)	3
49	41	*SCAPER*	c.625A>T, p.(Thr209Ser)	3
53	44	*CERKL*	c.847C>T, p.(Arg283*)	5
54	45	*CEP290*	c.1219_1220del, p.(Met407Glufs*14)	5
55	46	*RPGRIP1*	c.3570G>T, p.(Arg1190Ser)	3
		RP1	c.3946_3947delinsTT, p.(Ala1316Phe)	3
57	48	*AGBL5*	c.2500G>T, p.(Ala834Ser)	3
		RP1L1	c.791C>T, p.(Ser264Leu)	3
		PEX5	c.135_147+33delinsC	4
		ADGRV1	c.6229G>A, p.(Glu2077Lys)	3
		AHI1	c.1892G>A, p.(Arg631Gln)	3
58	49	*TTC21B*	c.1320del, p.(Phe440Leufs*4)	4

59	50	*RIMS1*	c.3203T>C, p.(Leu1068Pro)	3
		TREX1	c.341G>A, p.(Arg114His)	5
		USH2A	c.2299del, p.(Glu767Serfs*21)	5
		RPGRIP1	c.832del, p.(Arg278Aspfs*15)	4
